# Genetic counseling and testing in individuals at high risk for familial Alzheimer’s Disease from Latin America

**DOI:** 10.1002/alz.084526

**Published:** 2025-01-09

**Authors:** Jorge J. Llibre‐Guerra, Pablo Miguel Bagnati, Marisol Londoño Castaño, Maria L Fernandez, Beatriz E Mora‐Henao, Patricio Chrem Mendez, Luz E Varela Londoño, David Fernando Aguillon, Ezequiel Surace, Juan D Barbaran Manco, Carlos M Restrepo‐Fernández, Ellen Ziegemeier, Gabriela Vigo, Gabriel A Varga‐Cuadros, Eric McDade, Randall J. Bateman, Ricardo Allegri, Francisco Lopera

**Affiliations:** ^1^ Washington University in St. Louis, School of Medicine, St. Louis, MO USA; ^2^ Fleni, Buenos Aires Argentina; ^3^ Grupo de Neurociencias de Antioquia, Universidad de Antioquia, Medellin Colombia; ^4^ Fundación Médica de Enfermedades Raras, Medellín Colombia; ^5^ Grupo de Neurociencias de Antioquia, Universidad de Antioquia, Medellín Colombia; ^6^ Washington University in St. Louis School of Medicine, St. Louis, MO USA; ^7^ Hospital San Bernardo, Salta Argentina; ^8^ Washington University in St. Louis, St. Louis, MO USA; ^9^ Fleni, Buenos Aires, Buenos Aires Argentina

## Abstract

**Background:**

Genetic testing for individuals with dominantly inherited Alzheimer’s disease (DIAD) is now of greater relevance due to the existence of therapeutic trials available to this population. However, the impact and main drivers influencing the decision to seek genetic testing are relatively unknown in Latin America (LatAm). Here we present results from a regional genetic counseling and testing protocol implemented in LatAm.

**Method:**

Our aim was to investigate the psychosocial impact of genetic testing in asymptomatic individuals from families with DIAD. A non‐randomized, controlled, prospective observational trial was conducted. Asymptomatic, first‐degree relatives of patients with DIAD from families with a *presenilin 1*, pathogenic variant (p.I416T or p.M146L) were interviewed before and after genetic testing. Group allocation was done according to participants’ choice (non‐randomized). Participants who received their genetic status (GS) were allocated to the test group, while those who opted out were allocated to the observational group. The primary outcome included change from baseline in depression, general anxiety, and health‐related anxiety in the test group relative to the observational group, using linear mixed effects models and chi‐squared tests.

**Result:**

Forty‐five participants from Colombia and Argentina were invited and enrolled in the study. Thirty‐six individuals completed the genetic counseling sessions, and 25/36 (69.4%) learned their GS. Compared to families in Colombia, participants from Argentina were more likely to seek counseling and learn their GS (8/23, 34.9% vs, 17/20, 85.0%, respectively). Following genetic counseling, we found no significant differences between the group that learned their GS and those who did not learn their GS in anxiety (mean = 13.5, SD = 3.2 vs. 15.1, SD = 2.5, p = 0.2) or depression (mean = 5.4, SD = 2.3 vs. mean = 8.0, SD = 6.5, p = 0.6) scores. For both groups, anxiety and depression scores remained below cutoffs for clinical concern and without significant changes relative to baseline value levels.

**Conclusion:**

Our pilot study shows that a structured genetic counseling and testing protocol for individuals at‐risk of DIAD is safe. We found relevant cross‐country differences in willingness to learn genetic status, which may reflect differences in religion, educational level, and urban vs rural areas, among others. Future studies should include larger cohorts and expand to other countries in LatAm.

